# Context-sensitive computational mechanistic explanation in cognitive neuroscience

**DOI:** 10.3389/fpsyg.2022.903960

**Published:** 2022-07-22

**Authors:** Matthieu M. de Wit, Heath E. Matheson

**Affiliations:** ^1^Department of Neuroscience, Muhlenberg College, Allentown, PA, United States; ^2^Department of Psychology, University of Northern British Columbia, Prince George, BC, Canada

**Keywords:** cognitive neuroscience, degeneracy, functional brain mapping, levels of analysis, many-to-many mappings, mechanistic explanation, neural reuse, structure-function relationships

## Abstract

Mainstream cognitive neuroscience aims to build mechanistic explanations of behavior by mapping abilities described at the organismal level *via* the subpersonal level of computation onto specific brain networks. We provide an integrative review of these commitments and their mismatch with empirical research findings. Context-dependent neural tuning, neural reuse, degeneracy, plasticity, functional recovery, and the neural correlates of enculturated skills each show that there is a lack of stable mappings between organismal, computational, and neural levels of analysis. We furthermore highlight recent research suggesting that task context at the organismal level determines the dynamic parcellation of functional components at the neural level. Such instability prevents the establishment of specific computational descriptions of neural function, which remains a central goal of many brain mappers – including those who are sympathetic to the notion of many-to-many mappings between organismal and neural levels. This between-level instability presents a deep epistemological challenge and requires a reorientation of methodological and theoretical commitments within cognitive neuroscience. We demonstrate the need for change to brain mapping efforts in the face of instability if cognitive neuroscience is to maintain its central goal of constructing computational mechanistic explanations of behavior; we show that such explanations must be contextual at all levels.

## Introduction

### A brief history of the origins of modern functional brain mapping

The historical neuropsychological literature is full of case-studies of individuals with specific behavioral impairments following damage to local regions of the cerebral cortex. Perhaps most famously, Broca (1861) described a patient who, after damage to the left inferior frontal gyrus (IFG), permanently lost almost all of his expressive language abilities while his receptive abilities remained largely intact. Other patients may exhibit fluent (but incoherent) expressive language paired with receptive impairments, typically following lesions in the left superior temporal gyrus (STG). From these observations, Wernicke (1874/1969) developed one of the earliest neurocognitive models of language, which centered on the specific functions of IFG and STG and their connection *via* association fibers. Wernicke argued that complex functions such as language are the result of the interaction of multiple, simpler, sensory, motor, and association processes, thereby effectively ending the search for localized higher-level “faculties” in the brain that had dominated the study of brain-behavior relationships up to that time (Fancher, 1996; Bergeron, 2007). Since then, researchers have continued to grapple with understanding the functions of brain parts and their relationship to behavior, and one of the main goals of cognitive neuroscience remains mapping functional descriptions onto neural activity. But what does successful mapping look like?

### Functional brain mapping as the analysis, decomposition, and localization of function

The goal of mainstream cognitive neuroscience^1^ is to “investigate brain-behavior interactions” and to “address both descriptions of function and underlying brain events” (Journal of Cognitive Neuroscience, 2021); it seeks “an understanding how the functions of the physical brain can yield the thoughts, ideas, and beliefs” of the mind (Gazzaniga et al., 2019, p. 4). In the mainstream cognitive neuroscience research literature, the term *function* is critical to the enterprise. Importantly, function is often implicitly and interchangeably used at multiple, typically three, distinct levels of analysis (Marr, 1982/2010; Craver, 2014; Krakauer et al., 2017; Zednik, 2018; see Garson, 2016, for discussion of conceptualizations of biological function). The first level concerns that of observable behavior or its disorders. Here, cognitive neuroscientists distinguish between various complex behavioral or cognitive categories described at the personal or organismal level; that is, at the level of the behaving human being. For example, we talk about the abilities of (or impairments of) “paying attention,” “using a tool,” or “speaking.” These are the phenomena of which the discipline ultimately seeks a *mechanistic* explanation, in terms of a description of the parts and interactions of a system that gives rise to the phenomenon (Miłkowski, 2013; Craver and Tabery, 2015; Zednik, 2019).^2^

To this end, at the second, subpersonal, level the phenomenon described at the first level is decomposed into components that perform specific computational operations over representations and are organized together in a specific way (Bechtel and Abrahamsen, 2010). These components are therefore identified by their functions (Piccinini and Craver, 2011), the descriptions of which tend to be “human-interpretable” (Hasson et al., 2020). For instance, computational processes are hypothesized that implement, e.g., “attentional orienting,” “action selection,” or “linguistic retrieval,” which are *subcapacities* of the organismal-level capacity of paying attention, using a tool, and speaking, respectively. In other words, and following Wernicke’s lead, at the second level multiple latent, interacting, domain-specific or domain-general, functional components are postulated that explain the production of a particular complex behavior or cognitive ability observed at the first level. In general terms, it is widely accepted that computational operations are thought to involve inputs which feed into the manipulation of internal, typically – but not necessarily (Piccinini and Scarantino, 2011) – representational states to produce outputs that are then used downstream in further computational processes (Shea, 2018).

The third level at which function is invoked is that of the brain. Thus, following the characterization of a componential computational architecture at the second level (and sometimes *before* this characterization has taken place; see Agis and Hillis, 2017), an attempt is made to map the components onto the activity of the brain, effectively reifying that architecture; that is, the researcher tries to describe the coordinated computational operations in terms of physical neural processes (Piccinini and Shagrir, 2014; Burnston, 2021). There are at least two possible characterizations of this final step. Sometimes, the assumption is that a computational operation will directly map onto a specific brain “part,” where a part is broadly construed as a cell, localized assembly of cells or a distributed network of cells (Glasser et al., 2016). For instance, using lesion-symptom mapping or functional magnetic resonance imaging (fMRI), cognitive neuroscientists may search for a neural region or network of regions whose function it is to implement the computations that instantiate attentional orienting (while sometimes considering the biological constraints of the part such as, e.g., neural response latencies or receptor types; e.g., Kravitz et al., 2013), and another region or network whose function it is to implement the computations for action selection. However, sometimes the assumption is that a computational operation requires additional decomposition before it can be mapped onto the brain. For instance, using computational modeling of different types of cells or cell networks, attentional orienting is broken down further into component operations (e.g., a number of different computations in a connectionist model). Either way, the goal is to mechanistically explain organismal-level phenomena, *via* organized, interacting computational components, in terms of functionally specialized brain cells, assemblies, or networks that combine into interacting brain parts.^3^ Though cognitive neuroscience has moved on from structure-function mapping understood in the modular sense described earlier (e.g., expressive language is due to Broca’s area), many cognitive neuroscientists – implicitly or explicitly – still seek to discover *the* brain basis of our ability to pay attention, use a tool, or speak (Cabeza and Nyberg, 2000; see Zerilli, 2019 for a historical overview of this approach).

Given this, brain mapping continues to be a major effort, which can also be gleaned from its influence on the infrastructure of the field (e.g., the UCLA Brain Mapping Center; the journal *Human Brain Mapping*). Indeed, the idea of functional brain mapping is so deeply engrained in current thinking about brain-behavior relationships that it is implicit in our nomenclature and pervades textbooks and day-to-day public discourse about neuroscience. Thus, there is talk of the primary *visual* cortex or the dorsal *attention* network, which are believed to implement specialized computations underlying visual and attentional behavior, respectively. In a sense these efforts are clearly successful. For instance, it is possible to manipulate the activity of cortical regions using transcranial magnetic stimulation (TMS) and observe predictable effects on behavior, lesions to early visual regions result in predictable visual field deficits, and sophisticated computational models exist that impressively replicate various properties of real-world brain networks.

However, at the same time, the practice of functional brain mapping as defined above has met serious empirical and theoretical challenges, particularly in recent years (Uttal, 2001; Pessoa, 2008; Anderson’s, 2010, Anderson, 2014; Klein, 2012; Burnston, 2016b; Khalidi, 2017; Stanley et al., 2019; Viola, 2020; Zerilli, 2020). We are certainly not the first to note challenges in this domain: Previous neuroscientists critical of the functional brain mapping enterprise have called for a more effective integration of research findings at each level of analysis (Krakauer et al., 2017), have argued for a redescription of function at the level of local brain regions (Price and Friston, 2005; Poldrack, 2010), or have pointed out general limitations of current mapping efforts (Poldrack, 2006; Genon et al., 2018), among other criticisms. However, with some important exceptions to be discussed below, several of these researchers still aim to identify *the* componential computational operations of well-defined brain parts and in that sense maintain the central premises of functional brain mapping. For example, Shine et al. (2016, p. 26) state that “[i]n considering the computational capacities of independent brain regions, we will make the argument that computational specialization is not only abundant in the brain, but also that it would be difficult to imagine a working brain that did not contain such specialization.” However, the viability of this approach is in question.

### Outline of the review and further analysis

In contrast to previous critiques, we will suggest that the core problem cognitive neuroscience faces is an epistemological one: The present paper will integrate empirical and philosophical literature to show that the goal of giving *any* specific, computational description of *context-independently* defined brain parts is not possible, and therefore that the explanatory strategy of mainstream cognitive neuroscience is in need of revision. The section “Challenges to the Practice of Structure-Function Brain Mapping: An Integrative Review” provides an integrative review of the empirical literature on flexible neural tuning (section “Neural Tuning and Functional Brain Mapping”), plasticity and recovery of organismal-level function following brain lesions (section “Lesion Studies, Plasticity, and Functional Brain Mapping”), inter and intra-individual neural degeneracy (section “Degeneracy and Functional Brain Mapping”), neural reuse (section “Neural Reuse and Functional Brain Mapping”), and the neuroscience of enculturated skills such as reading and mathematics (section “Enculturated Skills and Functional Brain Mapping”). These areas are increasingly being investigated by researchers within cognitive neuroscience, and we summarize one consequence of these challenges that has been recognized in the field as “weak contextualism,” which denotes variable mappings relative to the organismal level of description (section “Consequences of Weak Contextualism”). However, section “Strong Contextualism, Instability of Mapping, and Indeterminate Part Ontology for Cognitive Neuroscience” describes the fundamental incommensurability of the goals of mechanistic explanation and any context-independent – even weak contextual – descriptions of brain function, and highlights a consequence not yet widely recognized within the field, a type of instability in functional mapping that reflects a “strong contextualism.” Most significantly, we review recent research that forces a reconsideration of what constitutes a relevant neural “part” to begin with and show that the parcellation of functional components shifts depending on the task context we choose to study at the organismal level. Finally, the section “Discussion: Implications for Cognitive Neuroscience” provides a brief methodological and theoretical sketch of a cognitive neuroscience that can maintain its central goal of constructing robust computational mechanistic explanations of behavior by being sensitive to the fact that such explanations must be contextual at all levels.

## Challenges to the practice of structure-function brain mapping: An integrative review

Below we provide an integrated review of research that present challenges to structure-function mapping (see Ames and Fiske, 2010; Anderson, 2014; Seifert et al., 2016; Hartwigsen, 2018; Rule et al., 2019 for previous focused reviews of each of the topics discussed in this section). Afterward, we will highlight the consequences this literature has had on the current state of the art in cognitive neuroscience.

### Neural tuning and functional brain mapping

Neuroscience has a long history of characterizing the neural tuning of brain regions, where manipulations of stimulus parameters of popular interest (e.g., line orientation, face-ness of an object, etc.) are correlated with changes in neural activity (firing rate, BOLD signal, etc.; see Buzsáki, 2020, for a brief description of this history). However, neuroimaging results show that many neural regions are not statically tuned to particular types of stimuli in a stable manner (Bair, 2005; Clopath et al., 2017). While flexibility in neural tuning at the single neuron level has been shown for some time (e.g., Miller, 2000), recent results show that the neural tuning of most brain regions appears capable of changing rapidly between different cognitive tasks. For instance, Çukur et al. (2013) had participants watch videos while lying in the scanner. They were instructed to attend to different features of the videos, specifically vehicles or humans. As they did so, it was shown that the tuning characteristics of almost every region in cortex shifted depending on the goal of the observer (i.e., voxel activity was better explained by responding to humans when searching for humans and vice versa for vehicles). Other research has demonstrated more specific effects. For instance, ventral cortical regions, typically implicated in identifying objects, do not do so in an all-or-none fashion but shift their response tuning to object identity depending on the exact task participants are performing (Harel et al., 2014). In addition, recent advancements in multi-voxel pattern analysis (MVPA; Kriegeskorte et al., 2008) suggest that the “representational geometry” (i.e., the abstract, multidimensional space of neural activity patterns; see Kriegeskorte and Diedrichsen, 2019) of different network nodes is dynamically defined by context. For instance, geometry of areas in the dorsal stream is better described by action similarity in a task where participants make judgments about action, but category similarity when participants make judgments about category (see also Anderson and Oates, 2010; Gallivan and Culham, 2015; Bracci et al., 2017). Similarly, patterns of activity in some areas of the cortex are implicitly tuned to the category of objects depending on cues that are available in the environment (Matheson et al., 2021). The important thing to note here is that the tuning of single cells determines the representational geometry that is measured in these studies (Kriegeskorte and Wei, 2021). Thus, changes in neural representational geometry in different tasks reflect changes in neural tuning across most of the cortex depending on the context.

Overall, these neural tuning findings challenge functional mapping efforts because they suggest that the target – the response properties of a neural region to particular stimulus information – is a moving one that is shaped by task context.

### Lesion studies, plasticity, and functional brain mapping

Second, findings of neural plasticity and recovery have long complicated the functional brain mapping literature. After (sometimes extensive, e.g., García et al., 2017; Bowren et al., 2021) brain damage, patients may be able to partially or fully recover the behavioral or cognitive ability that was lost (Kolb and Gibb, 2013). This trajectory of recovery can continue for years (Hartwigsen and Saur, 2019) and has even been observed following lesions in early sensory areas (in adult cats; see Jiang et al., 2021). One account of this phenomenon, consistent with the assumption that local neural regions perform specialized computational operations, is that the regained organismal-level function is supported by different computational processes. Perhaps a compensatory mechanism, involving different cognitive strategies, restores function at the organismal level (Dixon et al., 2008; see De Brigard, 2017 for a related discussion focusing on brain and cognitive strategy changes associated with healthy aging).

Another more commonly offered account of recovery that is consistent with functional brain mapping is that, as a result of plasticity, the remaining undamaged brain regions are able to reorganize themselves to accommodate new functions; that is, neural parts are repurposed to perform new computational operations. However, if recovery is indeed a matter of repurposing (i.e., the specific computational operation formerly realized by the damaged area is now performed by another area), one would expect to lose the function that the newly colonized area was responsible for. Alternatively, it is commonly postulated that the remaining intact tissue is utilized more efficiently after recovery, allowing for the former and the new computational operation to be implemented alongside each other, but this notion of redundancy (Friston and Price, 2003) begs the question why the colonized tissue was utilized less efficiently before, given that neural tissue is notoriously expensive to maintain. To our knowledge, observations of loss of one function accompanying recovery of another function appear to be largely absent from the patient literature.

On the contrary, several recent treatment studies have reported gains in the *language* domain as a result of *upper extremity* movement therapy in stroke patients (Harnish et al., 2014; Primaßin et al., 2015; see Anderlini et al., 2019 for review; and Stoll et al., 2021 for related work in limb apraxia). Furthermore, brain damage is often associated with multiple co-occurring deficits (e.g., patients presenting with both limb apraxia and aphasia following left hemisphere lesions), similarly suggesting that the impacted area supports diverse behavioral domains (Behrmann and Plaut, 2014; Goldenberg and Randerath, 2015). So called “crossed” cases of classical clusters of deficits have moreover been reported in patients with atypical lateralization of function (e.g., limb apraxia presenting together with aphasia following a *right* hemisphere lesion; Raymer, 1999; see Vingerhoets et al., 2013 for findings of co-lateralization in neurologically intact individuals). Each of these findings is hard to explain under the assumption that impacted functions are implemented by specialized neural parts, and may be interpreted as further evidence that the functional significance of any given region is sort of a chameleon which does not permit a context-free definition (Price et al., 2016; Price, 2018).^4^

### Degeneracy and functional brain mapping

It is increasingly recognized that the phenomenon of neural degeneracy – the notion that structurally different neural processes can produce equivalent behaviors at the organismal level – plays an important role in the brain (Edelman and Gally, 2001; Price and Friston, 2002; Noppeney et al., 2004, 2006; Figdor, 2010; Bateson and Gluckman, 2011; Sporns, 2011; Marder et al., 2015; Anderson, 2016; Seifert et al., 2016; De Brigard, 2017; Viola, 2020). Both inter- and intra-individual cases of degeneracy have been observed (Anderson, 2016). In the domain of numerical cognition, Tang et al. (2006) reported markedly different patterns of activation during simple arithmetic between native Chinese and native English speaking individuals, with left perisylvian activation in the former cultural group, and a network of “visual” and “premotor” regions in the latter, despite equivalent stimuli (Hindu-Arabic numerals) and equivalent performance at the behavioral level. In the language domain, Biduła et al. (2017) reported many different variants and degrees of language lateralization in neurologically intact individuals with normal language abilities, involving, in addition to more or less typical and atypical lateralization of classic language areas, right hemisphere components of the “default mode network” as well as an atypical role for the cerebellum. Finally, Merabet et al. (2008) provide evidence for intra-individual degeneracy in neurologically intact individuals by showing the existence of multiple, different neural substrates for braille reading (see De Brigard, 2017 for additional examples related to healthy aging). Degeneracy is clearly widespread, both in the intact and the lesioned brain (Fotopoulou, 2014; Price, 2018).^5^ Indeed, we would argue that functional recovery following brain damage can be considered a prime example of both inter and intra-individual degeneracy (see also Mogensen, 2011; Abrevaya et al., 2017; Hartwigsen and Saur, 2019).

### Neural reuse and functional brain mapping

Fourth, there is extensive evidence from neurologically intact individuals showing that some, if not most, brain regions are implicated in many different behaviors, suggesting that they can be reused in different contexts. That is, regions are typically capable of participating in a diverse array of functions. The above-mentioned study by Merabet et al. (2008) showed, using TMS, that the occipital (i.e., “visual”) cortex of blindfolded sighted participants became causally involved in tactile perception following an intense 5-day braille reading training program, providing evidence for its functional perceptual capacity beyond the visual modality (see also Murray et al., 2015). Similarly, in congenitally blind individuals who have never possessed sight, the occipital cortex has been shown to be sensitive to non-perceptual stimulus attributes such as the grammatical structure of spoken sentences and the difficulty of math equations (Bedny, 2017). Much evidence has been marshaled in the last decade or so showing that even gross functional distinctions at the organismal level, for instance the difference between emotional processes and cognitive ones (Pessoa, 2008) or between perception and action (Cisek, 2007), do not hold at the neural level, due (in part) to reuse.

The consistency of reports of functional heterogeneity suggests that reuse is not a curiosity but a general feature of the nervous system (Anderson, 2010, 2014). Importantly, despite empirical advancements showing that some regions can be functionally further subdivided (e.g., Broca’s area; Fedorenko and Blank, 2020) reuse is observed regardless of the level of granularity at which these analyses are performed (i.e., whether the brain was parsed into, say, 10 or a 1,000 regions); thus, reuse may be reduced (Poldrack, 2006) but does not go away at an increased spatial resolution (Anderson et al., 2013; Uddin et al., 2014). For instance, it is clear that, at the resolution of the entire brain, the entire brain is reused to support different behaviors, but neural reuse can be observed even at the resolution of single neurons, with neurons involved in either sensory or motoric functions depending on the behavioral and concomitant neural context in the roundworm *C. elegans* (Bargmann, 2012). The fact that reuse phenomena do not disappear at smaller resolutions presents a major challenge to determining structure-function mappings.

### Enculturated skills and functional brain mapping

Fifth and finally, Dehaene and Cohen (2007); (see Menary, 2015; Jones, 2018 for discussion) suggest that cognitive tasks that require enculturation and formal schooling in order to be displayed (like reading, writing, and mathematics) are supported by the neuronal “recycling” of neural regions that originally evolved for other purposes, yet have the right structure to implement those tasks – again a type of reuse, though reuse in this case is defined at longer timescales (see Borra and Luppino, 2018 for additional examples). The visual word form area in occipitotemporal cortex is a good example of such a region. The recycling account is convincing because these abilities have arguably emerged too recently (i.e., within the last few thousand years) for evolution to have generated specialized cortical regions to support them.

### Consequences of weak contextualism

This brief review integrates some of the most significant challenges facing cognitive neuroscience. Within the field, it is increasingly recognized that all of these phenomena suggest that the functional role of a brain region is context-dependent. However, while context-dependence is recognized by the field, we argue that this is recognition of a type of “weak” contextualism. By weak contextualism we mean that most researchers accept that behavioral context shapes a region/network’s functional role in organizing the organismal-level behavioral phenomenon, but still maintain that the functioning of the part itself is context-independent. That is, it is thought that *functions of brain parts are not stable when defined relative to the organismal level, but functions defined at the computational and neural level are* (compare to Burnston’s, 2016a,^2019^ “absolutism”). Researchers sympathize with weak contextualism when they make continued calls for context-independent computational descriptions of brain regions while recognizing that this computation implements a cognitive subcapacity that contributes to many different organismal-level behaviors. For example, Vingerhoets et al. (2013) report evidence in neurologically intact individuals that skilled action and expressive language involve strongly overlapping neural components. To account for this overlap, they postulate that these components implement the production of complex (i.e., precise, articulated, coordinated, nested) learned movement, an operation that is common to both tool use and speech, explaining its involvement in each of those contexts (see Knops et al., 2009; de Wit et al., 2012; Parkinson et al., 2014 for similar conceptualizations of shared informational or computational demands across different behavioral domains). Thus, with this approach, the functional description of neural parts is context-independent and is abstract enough to account for their contextual involvement in a wide range of tasks (Price and Friston, 2005; Shine et al., 2016; Humphreys et al., 2021; see also Anderson’s, 2010 early “working” vs. “use” conceptualization of neural reuse). Note that this type of contextualism, though now often accepted in the field, is already quite far removed from the traditional structure-function accounts described in the section “Introduction,” which have typically characterized the function of brain regions relative to the organismal level (e.g., the fusiform face area is important for face identification) or at a minimum in terms of cognitive subcapacities directly related to specific organismal-level abilities (e.g., a region involved in “attentional selection”). Regardless, an acceptance of weak contextualism would still allow us to find *the* computational function of a brain part (as was suggested in the case of Vingerhoets et al., 2013).

## Strong contextualism, instability of mapping, and indeterminate part ontology for cognitive neuroscience

While there may be sympathies toward weak contextualism within cognitive neuroscience, we argue that there is, inescapably, a form of “strong” contextualism that is not widely acknowledged (and therefore one that is far from accepted). Our argument builds on arguments within the philosophy of science regarding the consequences of seeking mechanistic explanation. Again, by mechanistic explanation, we mean the goal of providing a description of the parts and interactions of a system that give rise to a phenomenon (e.g., Craver, 2014), and in cognitive neuroscience the typical approach is to seek a mapping of behavior to computation to brain. Indeed, some philosophers have argued that a type of strong contextualism is an unavoidable consequence of seeking mechanistic explanations in general (i.e., not just a problem for cognitive neuroscience; see Lee and Dewhurst, 2021 for discussion) and therefore it is an unavoidable consequence of the explanatory goals of cognitive neuroscience. Here, we highlight two critical arguments to demonstrate strong contextualism; one regarding the functional mapping of the computational level to the brain level, and one regarding the parcellation of brain parts. We hope to show that these two epistemological issues demand methodological and theoretical re-orientation within the field that is much more significant than the demands of weak contextualism.

### Dynamic functional mappings

First, our integrative review leads to the conclusion that behavioral context not only determines the contribution of a brain part to the organization of behavior (i.e., weak contextualism), but that *context determines which computational operation that brain part implements* in support of the behavior. That is, the computation a region performs is not a specialization *of that region*, but rather is determined by the behavioral and neural context in which the region finds itself, and can shift when the context changes (Sporns, 2011; Klein, 2012; Anderson, 2014, 2015a; Burnston, 2016b,^2019^, ^2021^; Khalidi, 2017; and see Mesulam, 1990; McIntosh, 1999, 2000, 2004 for early arguments in this direction). Klein (2012) illustrates the idea clearly with a discussion of the function of pistons in trucks with engine brakes: “Most of the time, [pistons] compress a fuel-air mixture to the point of detonation and transmit the generated power to the crankshaft. On trucks equipped with engine brakes, the pistons also have a second function: when the engine brake is engaged, the pistons use power from the wheels to compress air in the cylinder, slowing the truck. *Which function the piston performs depends on things external to it*: whether it is powering or slowing the truck depends on the ignition system and the valve timing” (p. 955, italics added; in this metaphor, this is the neural context). Notice that the function of the piston depends on whether we are interested in explaining the “going” or “stopping” action of the truck (the behavioral context); specifically, it is causally transmitting explosive force to the crankshaft in one instance and is reacting to a vacuum in the cylinder in the other. Here, we have one part (the piston) that is not simply performing an abstract function useful to both stopping and going (cf. Vingerhoets et al., 2013), but that has a functional description that is dependent on our explanatory goals. Thus, under the strong contextualism view, there is nothing specialized about the functional role of a piston – there is *no* single computational operation performed by the part that plays a role across phenomena. Thus, the mapping of the part to the computation is unstable. We argue that this conclusion holds for brain parts. The empirical evidence collected in the last section is consistent with the idea that, when seeking mechanistic explanations, brain regions are best modeled with different computations in different tasks (regardless of whether the computations are described mathematically or verbally). For example, in one context a brain region’s activity might be best modeled as multiplying an input signal whereas in another it is best modeled with addition; in one context a network might be best described as an “integrator,” while in another it is a “filter,” etc. Thus, *functions of brain parts are not stable regardless of whether they are defined relative to the organismal, or computational, or neural level*.

Note that we are not denying that neurons (and neural networks) have physiological, morphological and other neuroanatomical (e.g., topological) characteristics that ensure they can do some things and not others, in the same way we wouldn’t deny a piston’s physical characteristics that allow it to do some things and not others. Our point is that a structure’s participation in any given behavior, while obviously – and importantly – constrained by its properties, is not determined by those properties, and we cannot describe a part’s functional contribution in a context-independent way *at any level* within our mechanistic explanation.

Importantly, not even extreme abstraction of the putative computational function will allow us to recover a stable structure-function mapping for a part. This is the case given that we can choose to study an infinite number of phenomena at the behavioral level and that we will evolve skills in the future that we have not characterized yet. For instance, while the “production of complex learned movement” attributed to a neural network may apply to explanations of skilled action and expressive language (Vingerhoets et al., 2013), we are unable to rule out that the same neural network supports some behavior that does not require “complex learned movement,” simply because we haven’t studied all existing behavioral phenomena, nor can we know what phenomena will arise in the future. Thus, unlike weak contextualism, strong contextualism reveals an instability in structure-function mapping that prevents us from ever finding *the* computational function of brain parts.

### Dynamic part ontologies

The second component of our argument relates to brain parts themselves. Because the identification of parts plays a central role in mechanistic explanation (Kaiser, 2018), identifying brain parts (defined as networks, anatomical regions, subregions, circuits, or single cells) is a cornerstone of cognitive neuroscience. Thus, a *central* issue in a cognitive neuroscience that takes mechanistic explanation seriously is determining the right “part ontology” (cf. Stanley et al., 2013; Viola and Zanin, 2017). However, strong contextualism shows that context determines not only a part’s computational role in any given behavior, but also determines what we should even consider to be a relevant part in the first place – that is, *context determines the appropriate ontology of parts* (cf. Poldrack and Yarkoni, 2016; Genon et al., 2018; Uddin et al., 2019). Strong contextualism challenges the idea that there is any context-independent parcellation of the brain that cognitive neuroscience can use in its mapping efforts. That is, changing our explanatory goals (i.e., which behavioral phenomenon we seek to explain) results in a redefining of the causally relevant parts that give rise to the phenomenon (see Craver and Kaplan, 2020). Indeed, it follows from the strong contextualism described here that (1) the boundaries of functionally relevant brain parts can shift every time we identify and want to explain a new behavioral phenomenon of interest and map the requisite computations onto brain parts, (2) these boundaries need not follow any obvious structural boundaries, and (3) they might shift even within participants, depending on context. These are not mere speculations. Consistent with point (2), functional boundaries within the brain need not follow any obvious neuroanatomical boundaries. For example, King et al. (2019) reported that functional subdivisions of the cerebellum did not coincide with lobular boundaries. Further, parcellation requires thresholding and clustering approaches, and it is well-known that different approaches will lead to different neuroanatomical maps (Sporns, 2012). Additionally, if, as we have argued above, the computational operation of a part is not context-independently predetermined by its material properties alone, then there is no reason to assume that its size, shape, or position *would* be context-independently definable for neural explanations of behavior. To see this, consider the following instructive metaphor in which a fictional researcher is interested in mapping function to structure in the extraneural human body. In describing high-fiving, the researcher identifies the hand as a whole as a functional part, while in describing the feeling of soft materials for pleasure, (parts of) individual finger segments constitute the relevant parts. In line with this analysis, and consistent with (1) and (3), Salehi et al. (2020) recently found that the boundaries of functional brain parts shift depending on the behavioral state of the participant. More specifically, they “demonstrate that the parcels are indeed consistent for a given condition, but reproducibly reconfigure across conditions, even when starting with the same initial atlas each time” (p. 2). This evidence shows that the foundational assumptions of cognitive neuroscience that it can map *the* brain parts to the computations that support organismal level behavior are not tenable.

Overall, strong contextualism presents an underappreciated characterization of brain-behavior interactions, revealing that context shifts are associated with instability of mapping between organismal, computational, and neural levels. It also leads to the counterintuitive conclusion that context determines part ontology (and not the reverse; see section “Discussion: Implications for Cognitive Neuroscience” below for more details). This epistemological consideration lays bare a unique challenge to cognitive neuroscience’s goal of “an understanding how the functions of the physical brain can yield the thoughts, ideas, and beliefs” of the mind (Gazzaniga et al., 2019, p. 4).

## Discussion: Implications for cognitive neuroscience

As discussed above, much of the current infrastructure of cognitive neuroscience has been and continues to be shaped by structure-functional brain mapping, and this has real consequences for how money, time, and physical resources get distributed, which includes how undergraduate and graduate students are introduced to the field and how knowledge is classified and disseminated. In our opinion, strong contextualism should force the field to seriously reconsider its approach to the study of how organismal-level functioning maps onto the computational level and how this maps onto the neural level. Below, we gesture toward a number of implications that researchers should consider when pursuing functional brain mapping.

Strong contextualism forces a shift in the goal of mainstream cognitive neuroscience toward developing relevant part ontologies and seeking computational mechanistic explanation *in context*. First, we must recognize that by deciding that we are interested in a particular organismal-level phenomenon in a particular situation (say, visual decision making in a dual rather than a single task setting), we “fix” the part ontology which best helps explain the behavior computationally – though of course we don’t know in advance what that part ontology is (in the same way that deciding whether we are interested in going or stopping helps fix the part ontology for the best explanation of truck behavior). Thus, cognitive neuroscience should abandon the aim of describing the context-independent specialized functions of well-defined brain parts. Rather, in specific contexts it should seek to answer the empirical question of what are *relatively stable parcellations*, likely at the level of the individual or at a minimum of a small subgroup of individuals, that together with context-dependent computational descriptions lead to robust explanations of the behavioral phenomena of interest (Salehi et al., 2020; Viola, 2020). That is, we switch from asking “What does neural part X do, and is it important for behavior Y?” to “Given behavior Y, what neural parts – maybe X, maybe Z – and associated putative computational functions are needed to explain it?”

Second, contrary to previous proposals that attempt to deal with weak contextualism (Price and Friston, 2005; Poldrack, 2010), our analysis suggests that the determination of a part ontology should actually take place at a relatively *low* (rather than high) level of abstraction, rendering it more useful for explanations in specific contexts (Klein, 2012; Anderson, 2014; Burnston, 2016b,^2021^). For instance, the hippocampus is a behaviorally promiscuous region that appears in explanations within many areas of cognitive neuroscience, most commonly in accounts of spatial or mnemonic behaviors (Jeffery, 2018, for a brief history) but other types of tasks as well, leading some to suggest there is an impasse in theorizing about hippocampus function (Ekstrom and Ranganath, 2018; see Humphreys et al., 2021 for a similar discussion centering on the angular gyrus). Consistent with weak contextualism, one approach to this staggering complexity has been to offer relatively abstract functional descriptions that could account for the hippocampus’s role in all of these phenomena, for instance pattern separation/completion (Yassa and Stark, 2011), scene construction (see Maguire et al., 2016 for debate), or context equivalency (Maurer and Nadel, 2021). Notice that these are all descriptions of functions that are abstract enough to be equally applicable across many parts of the brain and clearly show conceptual overlap (in at least the verbal accounts of their computational roles); further, it is hard to imagine identifying a behavioral phenomenon that would not implicate these types of computational functions. In contrast, strong contextualism points to why such functional promiscuity exists, and suggests that the hippocampus may be best understood, mechanistically, as a part that implements one type of computational operation that supports some behaviors in some tasks (e.g., memory in memory tasks), but other operations in other tasks (e.g., in navigation tasks). Importantly, the hippocampus, as a brain part defined in a context-independent way, may not be the right “part” for explanations of some other tasks which may require further decomposition into additional parts of the hippocampus itself (e.g., in imagination tasks). Another natural consequence from this analysis is that a functional part may crosscut gross neuroanatomical boundaries. As a hypothetical example, in some contexts but not others subregions of both the angular gyrus and the supramarginal gyrus may together constitute a functional part. Ultimately, the part ontology will depend on whether we are interested in explaining memory, navigation, or imagination.

Note that we are not denying that the hippocampus (and its neural contexts) have anatomical structure and physiological properties that we can characterize – and in fact, in moving away from focusing on context-independent functional descriptions, the description of such constraints takes on an increased importance (see also Anderson, 2015a; Bolt et al., 2017). For instance, there is evidence that, given its physiology, the hippocampus implements sequence generation (Buzsáki and Tingley, 2018). However, the question is about the context-independent relevance of any given anatomical or physiological feature for explanations within cognitive neuroscience. We are suggesting that the degree to which a particular part (e.g., hippocampus) with particular properties will make a stable appearance in computational *mechanistic explanations* of particular behaviors across contexts, and whether the mechanistic explanation (with a particular part ontology) results in strong predictions for behaviors of interest, is an empirical question to be addressed in cognitive neuroscience under the specter of strong contextualism.

Finally, the implications of strong contextualism extend beyond strategies for empirical work; it affects how we theorize and interpret results. Indeed, it is recognized by many that the behavioral and neural sciences are in a “theory crisis” (Eronen and Bringmann, 2021). Our review suggests that one source of the problem is failing to recognize the instability of mapping “paying attention,” “using a tool,” and “speaking” to computational operations and brain parts. Strong contextualism, then, suggests an additional way to address the theory crisis: By developing formal computational mechanistic explanations in context, cognitive neuroscience can explicitly test effective part ontologies with associated computational operations and functional roles based on the phenomena it seeks to understand, *where the phenomena it seeks to understand are open to revision and new classification*. Doing so opens up cognitive neuroscience not only to better “part ontologies” but also to more useful cognitive ontologies (Poldrack, 2010). Such an approach may lead to further advancements in which historic ontologies may be dismantled in favor of ones with potentially greater accuracy and hence hopefully wider reaching clinical and scientific impact (e.g., see Renoult et al., 2019, for an example of the dismantling of the episodic vs. semantic distinction and challenges this poses to conceptions of “memory”; see Anderson, 2011 for challenges to the reification of “attention”; see Buzsáki, 2020, for challenges to the distinction between perception and action, etc.). Thus, we foresee an iterative process in which respecting the notion that context fixes the part ontology that best explains behavior allows us to remain open to other functional mappings in other contexts, and searching for empirically useful part ontologies will also shape how we identify and characterize the behavioral phenomena we wish to explain (see also Anderson, 2015b). This is a suggestion that goes beyond stating we need to understand behavior better before we should seek mechanistic explanations (Krakauer et al., 2017); rather, it opens up the possibility of characterizing both part and behavioral ontology differently than we do now, making way for new theoretical approaches and insights into brain-behavior interactions in a way that allows cognitive neuroscience to feed back into the behavioral sciences.

We have encountered resistance to the arguments presented here. Strong contextualism is counterintuitive and there are two common reactions to these arguments that we want to briefly address before we conclude. First, one might accept weak contextualism but conclude the strong version is a step too far. Again, as we have shown, many researchers hold that there is a single abstract computational process that each region performs, and we just have to wait for technology and/or methodology to catch up before we can successfully map that region to a computation that maps to behavior. We have already addressed the limitations of this conclusion but want to highlight here that our arguments regarding strong contextualism are not technological or methodological, but primarily epistemological (and our integrative review provides empirical support for the epistemological issues we have highlighted). Advances in technology or methodology without theoretical advances will not address the problems identified in the sections “Challenges to the Practice of Structure-Function Brain Mapping: An Integrative Review” and “Strong Contextualism, Instability of Mapping, and Indeterminate Part Ontology for Cognitive Neuroscience.”

Second, one might argue that contextualized explanation is already the current state of the art, in which researchers in disconnected (siloed) subfields of cognitive neuroscience identify context-sensitive functions for brain parts, no one is *really* looking for context-independent explanations in cognitive neuroscience, and everyone already acknowledges that there is no principled way of discovering *the* mapping between a structure and its computations. If this was indeed the state of the art, there would be no reason for the anatomical parcellations to be similar across silos, as accepting strong contextualism means accepting empirically defined parcellations that will be contextually driven. However, parcellations *are* similar across silos (e.g., hippocampus is mainly structurally defined in both memory and navigation research), suggesting that researchers do think there is a meaningful context-independent parcellation of the brain that can be mapped to computations and behaviors of interest.

Ultimately, the efforts of cognitive neuroscience will be judged by their utility in fulfilling its goals as stated above (Gazzaniga et al., 2019; Journal of Cognitive Neuroscience, 2021). It is our contention that taking strong contextualism seriously, both when it comes to determining computational operations, and when it comes to determining the size, shape and location of the neural parts that instantiate these operations, will position us better to mechanistically explain the clinical behavioral observations that this article started with, and even to more fully understand what it means for us to pay attention, use a tool, or speak to one another.

## Author contributions

All authors listed have made a substantial, direct, and intellectual contribution to the work, and approved it for publication. Authors have contributed equally to this manuscript.

## Conflict of interest

The authors declare that the research was conducted in the absence of any commercial or financial relationships that could be construed as a potential conflict of interest.

## Publisher’s note

All claims expressed in this article are solely those of the authors and do not necessarily represent those of their affiliated organizations, or those of the publisher, the editors and the reviewers. Any product that may be evaluated in this article, or claim that may be made by its manufacturer, is not guaranteed or endorsed by the publisher.

## Footnotes

1 While cognitive neuroscience is currently dominated by cognitivist assumptions of representation, computation and mechanism (which we here call the mainstream approach), 4E (embodied, embedded, enactive, extended) as well as ecological approaches to the study of the brain are becoming increasingly prevalent. Further, recent work aims to merge key insights from both traditions (e.g., Piccinini, 2022). In the present article, we have chosen to limit our analysis of research practices as they occur in the mainstream cognitive neuroscience literature. A similar analysis of neuroscientific research practices within 4E and ecological frameworks would be a rather different enterprise and is outside the scope of the current article (for examples of work in this tradition, see Gibson, 1966; Chiel and Beer, 1997; Barrett, 2011; van Orden et al., 2012; Dotov, 2014; Anderson, 2014; Kiverstein and Miller, 2015; de Wit et al., 2017; Hutto et al., 2017; Dewhurst, 2018; Bruineberg and Rietveld, 2019; de Wit and Withagen, 2019; van der Weel et al., 2019; Ryan and Gallagher, 2020; Raja, 2021; Raja and Anderson, 2021).

2 The topic of how cognitive neuroscience has come to decide on which explananda are worth pursuing is interesting on its own, and a point we return to later, in the section “Discussion: Implications for Cognitive Neuroscience” (see Fancher, 1996; Kästner, 2017 for discussion). Further, more detailed discussion of the role of mechanism in science is beyond the scope of this manuscript (see Cartwright et al., 2020). Our argument assumes a commitment to understanding cognition mechanistically, that is, being explicable in terms of the joint action of parts. Even if one denies a specific characterization of mechanism as discussed in the philosophy of science, we think it uncontroversial that there is at least an implicit and informal understanding that cognitive neuroscience, like the cognitive psychology it inherits, is often seeking mechanistic explanation.

3 Note that different researchers may flesh out the second, computational level to a greater or lesser degree, and describe its connections to the third, neural level in more or less detail. Some researchers build full-fledged sophisticated computational models without paying much attention to the neural level, while other researchers attempt to characterize functions of brain regions using a combination of behavioral and neuroimaging methods, without fleshing out the computational operations of those regions in any detail.

4 A common alternative interpretation of the co-occurrence of symptoms is that lesions “do not color within the lines.” That is, that they may impact multiple – smaller – spatially co-located functionally specialized regions. However, this line of reasoning does not seem readily capable of accommodating the above-reported “crossed” cases and the impact of treatment in one behavioral domain on performance in another behavioral domain.

5 It is important to exclude the possibility that what seems like degeneracy at the neural level is in fact driven by organismal-level variability between or even within participants in cognitive or behavioral strategies when performing a task (Gardner et al., 2013; Berneiser et al., 2018).

## References

[B1] Abrevaya S. Sedeño L. Fitipaldi S. Pineda D. Lopera F. Buritica O. (2017). The road less traveled: alternative pathways for action-verb processing in parkinson’s disease. *J. Alzheimers Dis.* 55 1429–1435. 10.3233/JAD-160737 27834777

[B2] Agis D. Hillis A. E. (2017). The cart before the horse: when cognitive neuroscience precedes cognitive neuropsychology. *Cogn. Neuropsychol.* 34 420–429. 10.1080/02643294.2017.1314264 28562194 PMC6178233

[B3] Ames D. L. Fiske S. T. (2010). Cultural neuroscience. *Asian J. Soc. Psychol.* 13 72–82. 10.1111/j.1467-839X.2010.01301.x 23874143 PMC3714113

[B4] Anderlini D. Wallis G. Marinovic W. (2019). Language as a predictor of motor recovery: the case for a more global approach to stroke rehabilitation. *Neurorehabil. Neural Repair* 33 167–178. 10.1177/1545968319829454 30757952

[B5] Anderson B. (2011). There is no such thing as attention. *Front. Psychol.* 2:246. 10.3389/fpsyg.2011.00246 21977019 PMC3178817

[B6] Anderson M. L. (2010). Neural reuse: a fundamental organizational principle of the brain. *Behav. Brain Sci.* 33 245–66; discussion266–313. 20964882 10.1017/S0140525X10000853

[B7] Anderson M. L. (2014). *After Phrenology: Neural Reuse And The Interactive Brain.* Cambridge, MA: The MIT Press.

[B8] Anderson M. L. (2015a). Beyond componential constitution in the brain: starburst amacrine cells and enabling constraints. *Open MIND* 1 1–13. 10.15502/9783958570429

[B9] Anderson M. L. (2015b). Mining the brain for a new taxonomy of the mind. *Philos. Compass* 10 68–77. 10.1111/phc3.12155

[B10] Anderson M. L. (2016). “Neural reuse and in-principle limitations on reproducibility in cognitive neuroscience,” in *Reproducibility: Principles, Problems, Practices, and Prospects*, eds Atmanspacher H. Maasen S. (Hoboken, NJ: John Wiley and Sons, Ltd), 341–362. 10.1002/9781118865064.ch16

[B11] Anderson M. L. Oates T. (2010). A critique of multi-voxel pattern analysis. *Proc. Annu. Meet. Cogn. Sci. Soc.* 32 1511–1516.

[B12] Anderson M. L. Kinnison J. Pessoa L. (2013). Describing functional diversity of brain regions and brain networks. *NeuroImage* 73 50–58. 10.1016/j.neuroimage.2013.01.071 23396162 PMC3756684

[B13] Bair W. (2005). Visual receptive field organization. *Curr. Opin. Neurobiol.* 15 459–464. 10.1016/j.conb.2005.07.006 16023850

[B14] Bargmann C. I. (2012). Beyond the connectome: how neuromodulators shape neural circuits. *BioEssays* 34 458–465. 10.1002/bies.201100185 22396302

[B15] Barrett L. (2011). *Beyond The Brain: How Body And Environment Shape Animal And Human Minds.* Princeton, NJ: Princeton University Press.

[B16] Bateson P. P. G. Gluckman P. D. (2011). *Plasticity, Robustness, Development and Evolution.* Cambridge: Cambridge University Press.

[B17] Bechtel W. Abrahamsen A. (2010). Dynamic mechanistic explanation: computational modeling of circadian rhythms as an exemplar for cognitive science. *Stud. Hist. Philos. Sci. Part A* 41 321–333. 10.1016/j.shpsa.2010.07.003 21466124

[B18] Bedny M. (2017). Evidence from blindness for a cognitively pluripotent cortex. *Trends Cogn. Sci.* 21 637–648. 10.1016/j.tics.2017.06.003 28821345

[B19] Behrmann M. Plaut D. C. (2014). Bilateral hemispheric processing of words and faces: evidence from word impairments in prosopagnosia and face impairments in pure alexia. *Cereb. Cortex (New York, N.Y.: 1991)* 24 1102–1118. 10.1093/cercor/bhs390 23250954

[B20] Bergeron V. (2007). Anatomical and functional modularity in cognitive science: shifting the focus. *Philos. Psychol.* 20 175–195. 10.1080/09515080701197155

[B21] Berneiser J. Jahn G. Grothe M. Lotze M. (2018). From visual to motor strategies: training in mental rotation of hands. *NeuroImage* 167 247–255. 10.1016/j.neuroimage.2016.06.014 27321046

[B22] Biduła S. P. Przybylski Ł Pawlak M. A. Króliczak G. (2017). Unique neural characteristics of atypical lateralization of language in healthy individuals. *Front. Neurosci.* 11:525. 10.3389/fnins.2017.00525 28983238 PMC5613132

[B23] Bolt T. Anderson M. L. Uddin L. Q. (2017). Beyond the evoked/intrinsic neural process dichotomy. *Netw. Neurosci.* 2 1–22. 10.1162/NETN_a_00028 PMC5989985 29911670

[B24] Borra E. Luppino G. (2018). Large-scale temporo–parieto–frontal networks for motor and cognitive motor functions in the primate brain. *Cortex* 118 19–37. 10.1016/j.cortex.2018.09.024 30420100

[B25] Bowren M. D. Tranel D. Boes A. D. (2021). Preserved cognition after right hemispherectomy. *Neurol. Clin. Pract.* 11 e906–e908. 34992977 10.1212/CPJ.0000000000001015 PMC8723955

[B26] Bracci S. Daniels N. Op de Beeck H. (2017). Task context overrules object- and category-related representational content in the human parietal cortex. *Cereb. Cortex* 27 310–321. 10.1093/cercor/bhw419 28108492 PMC5939221

[B27] Broca P. (1861). Remarks on the seat of the faculty of articulated language, following an observation of aphemia (loss of speech). *Bull. Soc. Anat.* 6 330–357.

[B28] Bruineberg J. Rietveld E. (2019). What’s inside your head once you’ve figured out what your head’s inside of. *Ecol. Psychol.* 31 198–217. 10.1080/10407413.2019.1615204

[B29] Burnston D. C. (2016b). A contextualist approach to functional localization in the brain. *Biol. Philos.* 31 527–550. 10.1007/s10539-016-9526-2

[B30] Burnston D. C. (2016a). Computational neuroscience and localized neural function. *Synthese* 193 3741–3762. 10.1007/s11229-016-1099-8

[B31] Burnston D. C. (2019). Getting over atomism: functional decomposition in complex neural systems. *Br. J. Philos. Sci.* 72 743–772. 10.1093/bjps/axz039 32691291

[B32] Burnston D. C. (2021). Contents, vehicles, and complex data analysis in neuroscience. *Synthese* 199 1617–1639. 10.1016/j.neuroscience.2016.06.014 27316550 PMC4956474

[B33] Buzsáki G. (2020). The brain–cognitive behavior problem: a retrospective. *Eneuro* 7:ENEURO.0069-20.2020. 10.1523/ENEURO.0069-20.2020 32769166 PMC7415918

[B34] Buzsáki G. Tingley D. (2018). Space and time: the hippocampus as a sequence generator. *Trends Cogn. Sci.* 22 853–869. 30266146 10.1016/j.tics.2018.07.006 PMC6166479

[B35] Cabeza R. Nyberg L. (2000). Imaging cognition II: an empirical review of 275 pet and fMRI studies. *J. Cogn. Neurosci.* 12 1–47. 10.1162/08989290051137585 10769304

[B36] Cartwright N. Pemberton J. Wieten S. (2020). Mechanisms, laws and explanation. *Eur. J. Philos. Sci.* 10 1–19.

[B37] Chiel H. J. Beer R. D. (1997). The brain has a body: adaptive behavior emerges from interactions of nervous system, body and environment. *Trends Neurosci.* 20 553–557. 10.1016/S0166-2236(97)01149-1 9416664

[B38] Cisek P. (2007). Cortical mechanisms of action selection: the affordance competition hypothesis. *Philos. Trans. R. Soc. B* 362 1585–1599. 10.1098/rstb.2007.2054 17428779 PMC2440773

[B39] Clopath C. Bonhoeffer T. Hübener M. Rose T. (2017). Variance and invariance of neuronal long-term representations. *Philos. Trans. R. Soc. B* 372:20160161. 10.1098/rstb.2016.0161 28093555 PMC5247593

[B40] Craver C. F. (2014). “Levels,” in *Open Mind*, eds Metzinger T. Windt J. M. (Frankfurt am Main: MIND Group).

[B41] Craver C. F. Kaplan D. M. (2020). Are more details better? On the norms of completeness for mechanistic explanations. *Br. J. Philos. Sci.* 71 287–319.

[B42] Craver C. F. Tabery J. (2015). *Mechanisms In Science.* Available online at: https://stanford.library.sydney.edu.au/archives/sum2017/entries/science-mechanisms/ (accessed May 28, 2020).

[B43] Çukur T. Nishimoto S. Huth A. G. Gallant J. L. (2013). Attention during natural vision warps semantic representation across the human brain. *Nat. Neurosci.* 16 763–770. 10.1038/nn.3381 23603707 PMC3929490

[B44] De Brigard F. (2017). Cognitive systems and the changing brain. *Philos. Explor.* 20 224–241. 10.1080/13869795.2017.1312503

[B45] de Wit M. M. Withagen R. (2019). What should a “gibsonian neuroscience” look like? Introduction to the special issue. *Ecol. Psychol.* 31 147–151. 10.1080/10407413.2019.1615203

[B46] de Wit M. M. de Vries S. van der Kamp J. Withagen R. (2017). Affordances and neuroscience: steps towards a successful marriage. *Neurosci. Biobehav. Rev.* 80 622–629. 10.1016/j.neubiorev.2017.07.008 28757455

[B47] de Wit M. M. Van der Kamp J. Masters R. S. W. (2012). Distinct task-independent visual thresholds for egocentric and allocentric information pick up. *Conscious. Cogn.* 21 1410–1418. 10.1016/j.concog.2012.07.008 22868214

[B48] Dehaene S. Cohen L. (2007). Cultural recycling of cortical maps. *Neuron* 56 384–398. 10.1016/j.neuron.2007.10.004 17964253

[B49] Dewhurst J. (2018). Context-Sensitive ontologies for a non-reductionist cognitive neuroscience. *Aust. Philos. Rev.* 2 224–228. 10.1080/24740500.2018.1552102

[B50] Dixon R. A. Garrett D. D. Bäckman L. Stuss D. T. Winocur G. (2008). Principles of compensation in cognitive neuroscience and neurorehabilitation. *Cogn. Neurorehabil.* 2 22–38. 10.1080/09602011.2014.1003947 25609229 PMC4760110

[B51] Dotov D. G. (2014). Putting reins on the brain. How the body and environment use it. *Front. Hum. Neurosci.* 8:795. 10.3389/fnhum.2014.00795 25346675 PMC4191179

[B52] Edelman G. M. Gally J. A. (2001). Degeneracy and complexity in biological systems. *Proc. Natl. Acad. Sci. U.S.A.* 98 13763–13768. 10.1073/pnas.231499798 11698650 PMC61115

[B53] Ekstrom A. D. Ranganath C. (2018). Space, time, and episodic memory: the hippocampus is all over the cognitive map. *Hippocampus* 28 680–687. 28609014 10.1002/hipo.22750

[B54] Eronen M. I. Bringmann L. F. (2021). The theory crisis in psychology: how to move forward. *Perspect. Psychol. Sci.* 16 779–788. 10.1177/1745691620970586 33513314 PMC8273366

[B55] Fancher R. E. (1996). *Pioneers of Psychology*, 3rd Edn. London: Norton.

[B56] Fedorenko E. Blank I. A. (2020). Broca’s area is not a natural kind. *Trends Cogn. Sci.* 24 270–284. 10.1016/j.tics.2020.01.001 32160565 PMC7211504

[B57] Figdor C. (2010). Neuroscience and the multiple realization of cognitive functions. *Philos. Sci.* 77 419–456. 10.1086/652964

[B58] Fotopoulou A. (2014). Time to get rid of the ‘modular’ in neuropsychology: a unified theory of anosognosia as aberrant predictive coding. *J. Neuropsychol.* 8 1–19. 10.1111/jnp.12010 23469983

[B59] Friston K. J. Price C. J. (2003). Degeneracy and redundancy in cognitive anatomy. *Trends Cogn. Sci.* 7 151–152. 10.1016/S1364-6613(03)00054-8 12691761

[B60] Gallivan J. P. Culham J. C. (2015). Neural coding within human brain areas involved in actions. *Curr. Opin. Neurobiol.* 33 141–149. 10.1016/j.conb.2015.03.012 25876179

[B61] García A. M. Sedeño L. Herrera Murcia E. Couto B. Ibáñez A. (2017). A lesion-proof brain? Multidimensional sensorimotor, cognitive, and socio-affective preservation despite extensive damage in a stroke patient. *Front. Aging Neurosci.* 8:335. 10.3389/fnagi.2016.00335 28119603 PMC5222788

[B62] Gardner M. R. Brazier M. Edmonds C. J. Gronholm P. C. (2013). Strategy modulates spatial perspective-taking: evidence for dissociable disembodied and embodied routes. *Front. Hum. Neurosci.* 7:457. 10.3389/fnhum.2013.00457 23964229 PMC3741463

[B63] Garson J. (2016). *A Critical Overview of Biological Functions.* Cham: Springer International Publishing, 10.1007/978-3-319-32020-5

[B64] Gazzaniga M. S. Ivry R. B. Mangun G. R. (2019). *Cognitive Neuroscience: The Biology Of The Mind*, 5th Edn. New York, NY: W.W. Norton and Company.

[B65] Genon S. Reid A. Langner R. Amunts K. Eickhoff S. B. (2018). How to characterize the function of a brain region. *Trends Cogn. Sci.* 22 350–364. 10.1016/j.tics.2018.01.010 29501326 PMC7978486

[B66] Gibson J. J. (1966). *The Senses Considered As Perceptual Systems.* Boston, MA: Houghton Mifflin.

[B67] Glasser M. F. Coalson T. S. Robinson E. C. Hacker C. D. Harwell J. Yacoub E. (2016). A multi-modal parcellation of human cerebral cortex. *Nature* 536 171–178. 10.1038/nature18933 27437579 PMC4990127

[B68] Goldenberg G. Randerath J. (2015). Shared neural substrates of apraxia and aphasia. *Neuropsychologia* 75 40–49. 10.1016/j.neuropsychologia.2015.05.017 26004063

[B69] Harel A. Kravitz D. J. D. Baker C. I. C. (2014). Task context impacts visual object processing differentially across the cortex. *Proc. Natl. Acad. Sci. U.S.A.* 111 E962–E971. 10.1073/pnas.1312567111 24567402 PMC3956196

[B70] Harnish S. Meinzer M. Trinastic J. Fitzgerald D. Page S. (2014). Language changes coincide with motor and fMRI changes following upper extremity motor therapy for hemiparesis: a brief report. *Brain Imaging Behav.* 8 370–377. 10.1007/s11682-011-9139-y 21989635

[B71] Hartwigsen G. (2018). Flexible redistribution in cognitive networks. *Trends Cogn. Sci.* 22 687–698. 10.1016/j.tics.2018.05.008 29914734

[B72] Hartwigsen G. Saur D. (2019). Neuroimaging of stroke recovery from aphasia – Insights into plasticity of the human language network. *NeuroImage* 190 14–31. 10.1016/j.neuroimage.2017.11.056 29175498

[B73] Hasson U. Nastase S. A. Goldstein A. (2020). Direct fit to nature: an evolutionary perspective on biological and artificial neural networks. *Neuron* 105 416–434. 10.1016/j.neuron.2019.12.002 32027833 PMC7096172

[B74] Humphreys G. F. Ralph M. A. L. Simons J. S. (2021). A unifying account of angular gyrus contributions to episodic and semantic cognition. *Trends Neurosci.* 44 452–463. 10.1016/j.tins.2021.01.006 33612312

[B75] Hutto D. D. Peeters A. Segundo-Ortin M. (2017). Cognitive ontology in flux: the possibility of protean brains. *Philos. Explor.* 9795 1–17. 10.1080/13869795.2017.1312502

[B76] Jeffery K. J. (2018). The hippocampus: from memory, to map, to memory map. *Trends Neurosci.* 41 64–66. 29405927 10.1016/j.tins.2017.12.004

[B77] Jiang H. Stanford T. R. Rowland B. A. Stein B. E. (2021). Association cortex is essential to reverse hemianopia by multisensory training. *Cereb. Cortex* 31 5015–5023. 10.1093/cercor/bhab138 34056645 PMC8491673

[B78] Jones M. (2018). Numerals and neural reuse. *Synthese* 197 3657–3681. 10.1007/s11229-018-01922-y

[B79] Journal of Cognitive Neuroscience (2021). Available online at: https://direct.mit.edu/jocn [Accessed May 18, 2021].

[B80] Kaiser M. I. (2018). “The components and boundaries of mechanisms,” in *The Routledge Handbook of Mechanisms and Mechanical Philosophy*, eds Glennan S. Illari P. (New York, NY: Routledge).

[B81] Kästner L. (2017). *Philosophy of Cognitive Neuroscience: Causal Explanations, Mechanisms and Experimental Manipulations.* Berlin: De Gruyter.

[B82] Khalidi M. A. (2017). Crosscutting psycho-neural taxonomies: the case of episodic memory. *Philos. Explor.* 20 191–208. 10.1080/13869795.2017.1312501

[B83] King M. Hernandez-Castillo C. R. Poldrack R. A. Ivry R. B. Diedrichsen J. (2019). Functional boundaries in the human cerebellum revealed by a multi-domain task battery. *Nat. Neurosci.* 22 1371–1378. 10.1038/s41593-019-0436-x 31285616 PMC8312478

[B84] Kiverstein J. D. Miller M. (2015). The embodied brain: towards a radical embodied cognitive neuroscience. *Front. Hum. Neurosci.* 9:237. 10.3389/fnhum.2015.00237 25999836 PMC4422034

[B85] Klein C. (2012). Cognitive ontology and region- versus network-oriented analyses. *Philos. Sci.* 79 952–960. 10.1086/667843

[B86] Knops A. Thirion B. Hubbard E. M. Michel V. Dehaene S. (2009). Recruitment of an area involved in eye movements during mental arithmetic. *Science* 324 1583–1585. 10.1126/science.1171599 19423779

[B87] Kolb B. Gibb R. (2013). Searching for the principles of brain plasticity and behavior. *Cortex* 58 251–260. 24457097 10.1016/j.cortex.2013.11.012

[B88] Krakauer J. W. Ghazanfar A. A. Gomez-Marin A. Maciver M. A. Poeppel D. (2017). Neuroscience needs behavior: correcting a reductionist bias. *Neuron* 93 480–490. 10.1016/j.neuron.2016.12.041 28182904

[B89] Kravitz D. J. Saleem K. S. Baker C. I. Ungerleider L. G. Mishkin M. (2013). The ventral visual pathway: an expanded neural framework for the processing of object quality. *Trends Cogn. Sci.* 17 26–49. 10.1016/j.tics.2012.10.011 23265839 PMC3532569

[B90] Kriegeskorte N. Diedrichsen J. (2019). Peeling the onion of brain representations. *Annu. Rev. Neurosci.* 42 407–432. 31283895 10.1146/annurev-neuro-080317-061906

[B91] Kriegeskorte N. Wei X. X. (2021). Neural tuning and representational geometry. *arXiv* [Preprint]. arXiv: 2104.09743. 10.1038/s41583-021-00502-3 34522043

[B92] Kriegeskorte N. Mur M. Bandettini P. (2008). Representational similarity analysis—connecting the branches of systems neuroscience. *Front. Syst. Neurosci.* 2:4. 10.3389/neuro.06.004.2008 19104670 PMC2605405

[B93] Lee J. Dewhurst J. (2021). The mechanistic stance. *Eur. J. Philos. Sci.* 11 1–21.

[B94] Maguire E. A. Intraub H. Mullally S. L. (2016). Scenes, spaces, and memory traces: what does the hippocampus do? *Neuroscientist* 22 432–439. 26276163 10.1177/1073858415600389 PMC5021215

[B95] Marder E. Goeritz M. L. Otopalik A. G. (2015). Robust circuit rhythms in small circuits arise from variable circuit components and mechanisms. *Curr. Opin. Neurobiol.* 31 156–163. 10.1016/j.conb.2014.10.012 25460072 PMC4375070

[B96] Marr D. (1982/2010). *Vision: A Computational Investigation Into The Human Representation And Processing Of Visual Information.* Cambridge, MA: MIT Press.

[B97] Matheson H. E. Garcea F. E. Buxbaum L. J. (2021). Scene context shapes category representational geometry during processing of tools. *Cortex* 141 1–15. 10.1016/j.cortex.2021.03.021 34020166 PMC8319064

[B98] Maurer A. P. Nadel L. (2021). The continuity of context: a role for the hippocampus. *Trends Cogn. Sci.* 25 187–199. 33431287 10.1016/j.tics.2020.12.007 PMC9617208

[B99] McIntosh A. R. (1999). Mapping cognition to the brain through neural interactions. *Memory* 7 523–548. 10.1080/096582199387733 10659085

[B100] McIntosh A. R. (2000). Towards a network theory of cognition. *Neural Netw.* 13 861–870. 10.1016/S0893-6080(00)00059-9 11156197

[B101] McIntosh A. R. (2004). Contexts and catalysts: a resolution of the localization and integration of function in the brain. *Neuroinformatics* 2 175–182. 10.1385/NI:2:2:175 15319515

[B102] Menary R. (2015). “Mathematical cognition—a case of enculturation,” in *Open MIND*, eds Metzinger T. Windt J. M. (Frankfurt am Main: MIND Group), 25. 10.15502/9783958570818

[B103] Merabet L. B. Hamilton R. Schlaug G. Swisher J. D. Kiriakopoulos E. T. Pitskel N. B. (2008). Rapid and reversible recruitment of early visual cortex for touch. *PLoS One* 3:e3046. 10.1371/journal.pone.0003046 18728773 PMC2516172

[B104] Mesulam M.-M. (1990). Large-scale neurocognitive networks and distributed processing for attention, language, and memory. *Ann. Neurol.* 28 597–613. 10.1002/ana.410280502 2260847

[B105] Miłkowski M. (2013). *Explaining The Computational Mind.* Cambridge, MA: The MIT Press.

[B106] Miller E. K. (2000). The prefontral cortex and cognitive control. *Nat. Rev. Neurosci.* 1 59–65. 10.1038/35036228 11252769

[B107] Mogensen J. (2011). Reorganization of the injured brain: implications for studies of the neural substrate of cognition. *Front. Psychol.* 2:7. 10.3389/fpsyg.2011.00007 21713186 PMC3111425

[B108] Murray M. M. Thelen A. Thut G. Romei V. Martuzzi R. Matusz P. J. (2015). The multisensory function of the human primary visual cortex. *Neuropsychologia* 1 1–9. 10.1016/j.neuropsychologia.2015.08.011 26275965

[B109] Noppeney U. Friston K. J. Price C. J. (2004). Degenerate neuronal systems sustaining cognitive functions. *J. Anat.* 205 433–442. 10.1111/j.0021-8782.2004.00343.x 15610392 PMC1571441

[B110] Noppeney U. Penny W. D. Price C. J. Flandin G. Friston K. J. (2006). Identification of degenerate neuronal systems based on intersubject variability. *NeuroImage* 30 885–890. 10.1016/j.neuroimage.2005.10.010 16300969

[B111] Parkinson C. Liu S. Wheatley T. (2014). A common cortical metric for spatial, temporal, and social distance. *J. Neurosci.* 34 1979–1987. 10.1523/JNEUROSCI.2159-13.2014 24478377 PMC6827593

[B112] Pessoa L. (2008). On the relationship between emotion and cognition. *Nat. Rev. Neurosci.* 9 148–158. 18209732 10.1038/nrn2317

[B113] Piccinini G. (2022). Situated neural representations: solving the problems of content. *Front. Neurorobotics* 16:846979. 10.3389/fnbot.2022.846979 35496901 PMC9049929

[B114] Piccinini G. Craver C. F. (2011). Integrating psychology and neuroscience: functional analyses as mechanism sketches. *Synthese* 183 283–311. 10.1007/s11229-011-9898-4

[B115] Piccinini G. Scarantino A. (2011). Information processing, computation, and cognition. *J. Biol. Phys.* 37 1–38. 10.1007/s10867-010-9195-3 22210958 PMC3006465

[B116] Piccinini G. Shagrir O. (2014). Foundations of computational neuroscience. *Curr. Opin. Neurobiol.* 25 25–30. 10.1016/j.conb.2013.10.005 24709597

[B117] Poldrack R. A. (2006). Can cognitive processes be inferred from neuroimaging data? *Trends Cogn. Sci.* 10 59–63. 10.1016/j.tics.2005.12.004 16406760

[B118] Poldrack R. A. (2010). Mapping mental function to brain structure: how can cognitive neuroimaging succeed? *Perspect. Psychol. Sci.* 5 753–761. 10.1177/1745691610388777 25076977 PMC4112478

[B119] Poldrack R. A. Yarkoni T. (2016). From brain maps to cognitive ontologies: informatics and the search for mental structure. *Annu. Rev. Psychol.* 67 1–26. 10.1146/annurev-psych-122414-033729 26393866 PMC4701616

[B120] Price C. J. (2018). The evolution of cognitive models: from neuropsychology to neuroimaging and back. *Cortex* 107 37–49. 10.1016/j.cortex.2017.12.020 29373117 PMC5924872

[B121] Price C. J. Friston K. J. (2002). Degeneracy and cognitive anatomy. *Trends Cogn. Sci.* 6 416–421. 10.1016/S1364-6613(02)01976-9 12413574

[B122] Price C. J. Friston K. J. (2005). Functional ontologies for cognition: the systematic definition of structure and function. *Cogn. Neuropsychol.* 22 262–275. 10.1080/02643290442000095 21038249

[B123] Price C. J. Hope T. T. M. Seghier M. L. (2016). Ten problems and solutions when predicting individual outcome from lesion site after stroke. *NeuroImage* 145 200–208. 10.1016/j.neuroimage.2016.08.006 27502048 PMC5154378

[B124] Primaßin A. Scholtes N. Heim S. Huber W. Binkofski F. Werner C. J. (2015). Determinants of concurrent motor and language recovery during intensive therapy in chronic stroke patients: four single-case studies. *Front. Neurol.* 6:215. 10.3389/fneur.2015.00215 26500606 PMC4598579

[B125] Raja V. (2021). Resonance and radical embodiment. *Synthese* 199 113–141. 10.1007/s11229-020-02610-6

[B126] Raja V. Anderson M. L. (2021). “Behavior considered as an enabling constraint,” in *Neural Mechanisms*, Vol. 17 eds Calzavarini F. Viola M. (Cham: Springer International Publishing), 209–232. 10.1007/978-3-030-54092-0_10

[B127] Raymer A. M. (1999). Crossed apraxia: implications for handedness. *Cortex* 35 183–199. 10.1016/S0010-9452(08)70793-7 10369092

[B128] Renoult L. Irish M. Moscovitch M. Rugg M. D. (2019). From knowing to remembering: the semantic–episodic distinction. *Trends Cogn. Sci.* 23 1041–1057. 31672430 10.1016/j.tics.2019.09.008

[B129] Rule M. E. O’Leary T. Harvey C. D. (2019). Causes and consequences of representational drift. *Curr. Opin. Neurobiol.* 58 141–147. 31569062 10.1016/j.conb.2019.08.005 PMC7385530

[B130] Ryan K. J. J. Gallagher S. (2020). Between ecological psychology and enactivism: is there resonance? *Front. Psychol.* 11:1147. 10.3389/fpsyg.2020.01147 32581956 PMC7283906

[B131] Salehi M. Greene A. S. Karbasi A. Shen X. Scheinost D. Constable R. T. (2020). There is no single functional atlas even for a single individual: functional parcel definitions change with task. *NeuroImage* 208:116366. 10.1016/j.neuroimage.2019.116366 31740342

[B132] Seifert L. Komar J. Araújo D. Davids K. (2016). Neurobiological degeneracy: a key property for functional adaptations of perception and action to constraints. *Neurosci. Biobehav. Rev.* 69 159–165. 10.1016/j.neubiorev.2016.08.006 27506266

[B133] Shea N. (2018). *Representation in Cognitive Science.* Oxford: Oxford University Press.

[B134] Shine J. M. Eisenberg I. Poldrack R. A. (2016). Computational specificity in the human brain. *Behav. Brain Sci.* 39:e131. 10.1017/S0140525X1500165X 27562637

[B135] Sporns O. (2011). *Networks Of The Brain.* Cambridge, MA: The MIT Press.

[B136] Sporns O. (2012). *Discovering The Human Connectome.* Cambridge, MA: MIT Press.

[B137] Stanley M. L. Gessell B. De Brigard F. (2019). Network modularity as a foundation for neural reuse. *Philos. Sci.* 86 23–46. 10.1086/701037

[B138] Stanley M. L. Moussa M. N. Paolini B. Lyday R. G. Burdette J. H. Laurienti P. J. (2013). Defining nodes in complex brain networks. *Front. Comput. Neurosci.* 7:169. 10.3389/fncom.2013.00169 24319426 PMC3837224

[B139] Stoll H. de Wit M. M. Middleton E. L. Buxbaum L. J. (2021). Treating limb apraxia *via* action semantics: a preliminary study. *Neuropsychol. Rehabil.* 31 1145–1162. 10.1080/09602011.2020.1762672 32429797 PMC7674248

[B140] Tang Y. Zhang W. Chen K. Feng S. Ji Y. Shen J. (2006). Arithmetic processing in the brain shaped by cultures. *Proc. Natl. Acad. Sci. U.S.A.* 103 10775–10780. 10.1073/pnas.0604416103 16815966 PMC1502307

[B141] Uddin L. Q. Kinnison J. Pessoa L. Anderson M. L. (2014). Beyond the tripartite cognition—emotion—interoception model of the human insular cortex. *J. Cogn. Neurosci.* 26 16–27. 10.1162/jocn_a_00462 23937691 PMC4074004

[B142] Uddin L. Q. Yeo B. T. T. Spreng R. N. (2019). Towards a universal taxonomy of macro-scale functional human brain networks. *Brain Topogr.* 32 926–942. 10.1007/s10548-019-00744-6 31707621 PMC7325607

[B143] Uttal W. R. (2001). *The New Phrenology: The Limits Of Localizing Cognitive Processes In The Brain.* Cambridge, MA: MIT Press.

[B144] van der Weel F. R. Agyei S. B. van der Meer A. L. H. (2019). Infants’ brain responses to looming danger: degeneracy of neural connectivity patterns. *Ecol. Psychol.* 31 182–197. 10.1080/10407413.2019.1615210

[B145] van Orden G. Hollis G. Wallot S. (2012). The blue-collar brain. *Front. Physiol.* 3:207. 10.3389/fphys.2012.00207 22719730 PMC3376425

[B146] Vingerhoets G. Alderweireldt A. S. Vandemaele P. Cai Q. Van der Haegen L. Brysbaert M. (2013). Praxis and language are linked: evidence from co-lateralization in individuals with atypical language dominance. *Cortex* 49 172–183. 10.1016/j.cortex.2011.11.003 22172977

[B147] Viola M. (2020). Beyond the platonic brain: facing the challenge of individual differences in function-structure mapping. *Synthese* 199 2129–2155. 10.1007/s11229-020-02875-x

[B148] Viola M. Zanin E. (2017). The standard ontological framework of cognitive neuroscience: some lessons from broca’s area. *Philos. Psychol.* 5089 1–25. 10.1080/09515089.2017.1322193

[B149] Wernicke C. (1874/1969). “The symptom complex of aphasia,” in *Proceedings of the Boston Colloquium for the Philosophy of Science 1966/1968*, eds Cohen R. S. Wartofsky M. W. (Dordrecht: Springer Netherlands), 34–97. 10.1007/978-94-010-3378-7_2

[B150] Yassa M. A. Stark C. E. (2011). Pattern separation in the hippocampus. *Trends Neurosci.* 34 515–525. 21788086 10.1016/j.tins.2011.06.006 PMC3183227

[B151] Zednik C. (2018). “Mechanisms in cognitive science,” in *The Routledge Handbook of Mechanisms and Mechanical Philosophy*, eds Illari P. M. Glennan S. (London: Routledge), 389–400.

[B152] Zednik C. (2019). “Computational cognitive neuroscience,” in *The Routledge Handbook of the Computational Mind*, eds Sprevak M. Colombo M. (Abingdon: Routledge).

[B153] Zerilli J. (2019). Neural reuse and the modularity of mind: where to next for modularity? *Biol. Theory* 14 1–20. 10.1007/s13752-018-0309-7

[B154] Zerilli J. (2020). *The Adaptable Mind: What Neuroplasticity and Neural Reuse Tells Us About Language and Cognition.* Oxford: Oxford University Press.

